# Factors hindering coverage of targeted mass treatment with primaquine in a malarious township of northern Myanmar in 2019–2020

**DOI:** 10.1038/s41598-023-32371-4

**Published:** 2023-04-12

**Authors:** Pyae Linn Aung, Myat Thu Soe, Than Naing Soe, Thit Lwin Oo, Kyawt Mon Win, Liwang Cui, Myat Phone Kyaw, Jetsumon Sattabongkot, Kamolnetr Okanurak, Daniel M. Parker

**Affiliations:** 1 Myanmar Health Network Organization, Yangon, Myanmar; 2 Mahidol Vivax Research Unit, Faculty of Tropical Medicine, Mahidol University, Bangkok, Thailand; 3 Department of Public Health, Ministry of Health, NayPyiTaw, Myanmar; 4 Division of Infectious Diseases and International Medicine, Department of Internal Medicine, Morsani College of Medicine, University of South Florida, 3720 Spectrum Boulevard, Suite 304, Tampa, FL 33612 USA; 5 Department of Social and Environmental Health, Faculty of Tropical Medicine, Mahidol University, Bangkok, Thailand; 6 Department of Population Health and Disease Prevention, Department of Epidemiology, University of California, Irvine, USA

**Keywords:** Disease prevention, Health services, Public health

## Abstract

Targeted mass primaquine treatment (TPT) might be an effective intervention to facilitate elimination of vivax malaria in Myanmar by 2030. In this study, we explored the factors hindering coverage of a TPT campaign conducted in a malarious township of northern Myanmar. From August 2019 to July 2020, a cross-sectional exploratory design including quantitative and qualitative data was conducted in five villages with high *P. vivax* prevalence following a TPT campaign. Among a targeted population of 2322; 1973 (85.0%) participated in the baseline mass blood survey (MBS) and only 52.0% of the total targeted population (1208, 91.9% of total eligible population) completed the TPT. G6PD deficiency was found among 13.5% of total MBS participants and those were excluded from TPT. Of 1315 eligible samples, farmers and gold miners, males, and those aged 15 to 45 years had higher percentages of non-participation in TPT. Qualitative findings showed that most of the non-participation groups were outside the villages during TPT because of time-sensitive agricultural and other occupational or education-related purposes. In addition to mitigating of some inclusion criteria (i.e. including young children or offering weekly PQ treatment to G6PD deficient individuals), strengthening community awareness and increasing engagement should be pursued to increase community participation.

## Introduction

Malaria remains a priority public health concern in the Southeast Asia (SEA) Region. The region has the second highest malaria burden in the world, following Africa. Among 11 member countries from SEA, Indonesia, India and Myanmar are the top three most malaria endemic countries^1^. There have been remarkable improvements in the malaria burden in Myanmar over the last decade since the country committed to the goal of being malaria-free by 2030^2^. Still, in 2020 there were 58,836 confirmed malaria cases and ten reported malaria deaths in Myanmar^1^. The trend suggested an 88.3% morbidity reduction compared to 2010 data^1^. The species composition has likewise been shifting during this same time period. In 2011, *P. vivax* accounted for only 46.0% of all infections but by 2020 it composed 74.2% of all cases^1^. The reported *P. vivax* cases likely include many relapse cases, either because of poor compliance with a 14-day primaquine (PQ) treatment or lack of PQ administration in case of contraindication (e.g. pregnant women)^3^. Relapse cases need to be addressed as they can lead to transmission and while most elimination efforts have focused on falciparum malaria, it will be necessary to focus on *P. vivax* as well in order to achieve malaria elimination^2^. The existence of latent *P. vivax* reservoirs therefore poses a threat hindering the achievement of malaria elimination^4,5^.

The National Malaria Treatment Guidelines (NMTG)^6^ were endorsed in Myanmar in 2015 and have been regularly updated according to the latest global guidelines. The NMTG provide detailed information for using 14-day PQ (0.25 mg/kg bodyweight) as radical cure for *P. vivax* infections. Worldwide, PQ is widely used, and several studies have shown its efficacy^3,7–9^. Previous studies have suggested that many reported *P. vivax* cases were relapses rather than new infections^10–12^. The latent stage hypnozoites in the human liver can reactivate, leading to clinical relapse weeks or months after getting the infection^13^. Radical cure with PQ can clear hypnozoites^6^, however the long duration of the 14-day regimen has been difficult with regard to adherence^14^. Shorter courses of PQ, or new hypnozoitocidal drugs like tafenoquine that only require a single dose, might lead to more effective outcomes but must also be balanced with the dangers of hemolysis in G6PD deficient patients (especially in Myanmar where G6PD testing is not done prior to treatment)^6,15^. G6PD deficiency is an inherited, X-linked trait. A recent study in western Myanmar^16^ reported an approximate 10.0% prevalence of G6PD deficiency among malaria infected individuals. While there is diversity in G6PD variants, the most common variant from Myanmar has been the Mahidol variant or 1311 T/93C haplotype^16,17^. Currently the WHO recommends the 14-day PQ regimen^18^ and this recommendation has been reflected in the NMTG. Meanwhile, a straightforward tool that can detect the presence of hypnozoites remains unavailable, G6PD deficiency testing remains difficult in normal field settings, G6PD deficiency is high in some malarious settings, and adherence to the full 14-day regimen of PQ is a continuing challenge.

Large-scale mass primaquine treatment is one approach to address reservoirs of latent *P. vivax* parasites^19,20^. Targeted mass primaquine treatment (TPT) has been successful in eliminating temperate-zone *P. vivax* in China^21^. Studies on TPT to eliminate *P. vivax* in China^21^ and the Democratic People’s Republic of Korea^22^ pointed out the importance of achieving high coverage and being aware of a greater risk of hemolysis among patients with G6PD deficiency. Still, the feasibility of eliminating *P. vivax* from tropical and subtropical areas like the Greater Mekong Subregion (GMS), where G6PD deficiency is highly prevalent, has not been evaluated^23^. Moreover, the current NMTG^6^ also lack information about the implementation of TPT activities. Therefore, a TPT study was conducted in a township in Myanmar from 2019 to 2020 to assess the feasibility of using this elimination focused tool in this setting^24^. The TPT campaign included mass administration of PQ targeted specifically to people without G6PD deficiency and living in *P. vivax* high burden villages^24^.

The National Malaria Strategic Plan^4^ described the use of active surveillance through mass blood surveys (MBS) in active malaria foci to determine the point prevalence at a time or to fill the gap of the routine passive case detection coverage. Furthermore, MBS should be paired with TPT activities to evaluate potential changes in malaria prevalence before and after TPT^22^. Blood tests including G6PD investigations and monitoring Hb% must also be done prior to and during the mass treatment activities.

The relative success of mass drug administration is dependent on several factors, including transmission level, the antimalarial used, and coverage of the targeted population (linked to community participation)^19^. Historically, mass drug administration campaigns with low coverage have shown minimal effectiveness. Many campaigns seek to achieve a coverage of at least 80.0%, especially for falciparum malaria but also for vivax^19,25^. Particularly with regard to TPT for *P. vivax*, contraindications to PQ and general compliance with the full 14-day antimalarial regime are major challenges to achieving high population coverage^22^. Next, it is also important to address the challenges as well as the needs of the community to participate in a mass treatment activity. Therefore, this study aimed to document the factors hindering people's participation in a recent targeted mass treatment trial with primaquine in northern Myanmar.

## Methods

### Study design

The study incorporated a cross-sectional, mixed methods design including both quantitative and qualitative data collection among people living in a *P. vivax* endemic township in northern Myanmar from August 2019 to July 2020.

### Study site

**Figure 1:**
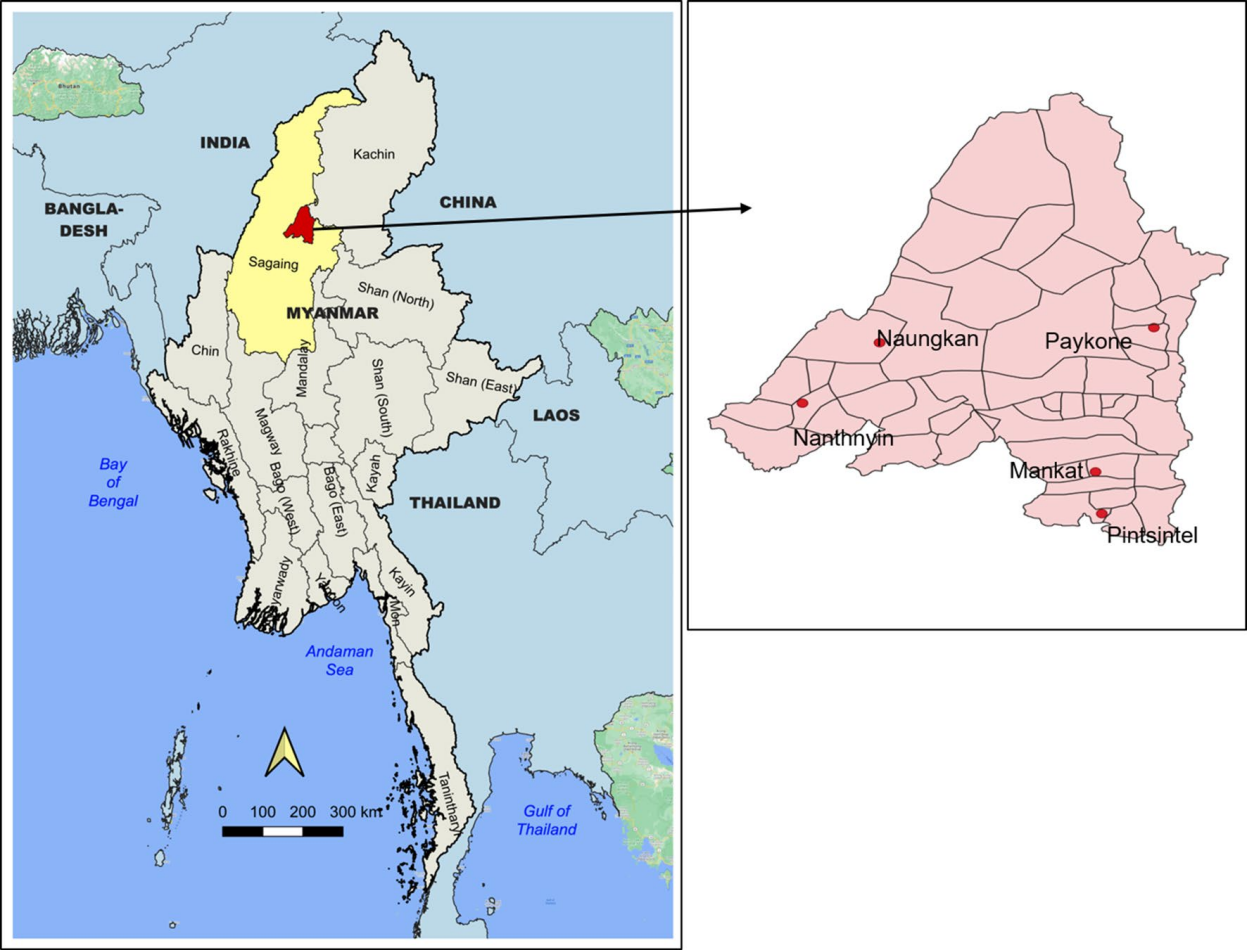
Location of study villages in Banmauk Township, Sagaing Region. The maps were created using Quantum GIS (QGIS) version 3.22 (https://www.qgis.org/en/site/forusers/download.html).

Myanmar is composed of 14 states and regions and one union territory. Among them, Sagaing is one of the highest malaria burden regions in northern Myanmar. Of 34 total townships in Sagaing, Banmauk township (Fig. 1) was purposively selected as a study township because of its high malaria prevalence. The overall township’s annual parasite incidence for all malaria was 11 and 3.8 per 1000 people at risk in 2017 and 2018, respectively^26^. Malaria diagnosis and treatment services are provided free of charge through the government basic health staff and village malaria volunteers. Currently, patients with *P. vivax* are treated according to the NMTG^6^ without G6PD testing and using Directly Observed Treatment (DOT).

Among a total of 219 villages from Banmauk, ten villages with the highest reported number of *P. vivax* cases were listed based on the 2018 reported malaria data from the township vector borne disease control (VBDC) team. Then from these ten villages, five villages (NantHnyin, NaungKan, MankKat, PayKone and PintSinTe) were randomly selected and targeted for a study on mass treatment with PQ (Fig. 1).

### Study population

In the selected five villages, there is a total population of 2322. The township has an estimated population of 110,000 consisting of Shan, Kadu, Kanan, and Burmese ethnic groups. Most people rely on agricultural-related work followed by gold mining in some areas. Population migration from some adjacent townships is also likewise possible. However, there are no goldmines in the immediate vicinity of the study villages though some people worked at the goldmines or forest-related settings in other areas and returned to the villages routinely. Nevertheless, detailed data on population movements were not available. In a preliminary study^24^, we found that more than 90.0% of study respondents agreed to participate in the TPT.

### Data collection

Both quantitative and qualitative data were collected in this study.

For the quantitative component, a baseline population census was conducted following village-level advocacy meetings in the five study villages in August 2019. The collected data included age, gender, occupation, ethnicity, education level, annual family income and pregnancy and breastfeeding statuses in the case of reproductive age women. Since then, a master file of population listing has been maintained.

All the residents over 1 year old from five selected villages were targeted for inclusion in the baseline MBS (mass blood survey). Conversely, children who were < 7 years old, pregnant women or breastfeeding mothers, people who were G6PD deficient, and people with Hb levels less than 8 g/dl were all excluded from TPT. During MBS, malaria microscopy as well as blood spots on filter papers for PCR analysis were also collected to detect malaria parasites. Study participants were included in the study regardless of their infection status. Those who were found to be malaria positive were prescribed schizonticidal drugs followed by the TPT regimen.

For G6PD testing, the CareStart G6PD RDT was used. It is a qualitative point-of-care visual screening test that identifies G6PD deficient patients using a whole blood sample. It uses the visual dye-colorization assay method and has a result time of 10 min. According to a test performance evaluation in Myanmar^27^, the G6PD kit showed almost 95.0% sensitivity and 90.0% specificity. However, to cover the possible false negative results by RDT, all the participants were crosschecked for the presence of dark colored urine during 14-day PQ course by the research team. No participants reported presence of dark colored urine during the 14-day PQ treatment.

Those eligible for TPT were followed up with a checklist by health officers to monitor the completeness of DOT and to monitor possible side effects of the daily PQ regimen. To find out the reasons for not being present in the villages during TPT, a simple question was asked to other family members about why those missed samples were away from the village.

After the TPT operation, qualitative data were collected through face-to-face in-depth interviews using interview guidelines. The guideline covered whether respondents knew about the implementation of TPT in the villages and the main reasons for not being included in the treatment campaign entirely or partially. The qualitative data also covered the reasons for not participating in the TPT. People from the five study villages were categorized into TPT and non-TPT groups. Individuals who completed the 14-day PQ regime were defined as the TPT group and those who were totally absent from any TPT activity including the initial MBS were defined as the non-MBS group. Those who were involved in MBS and were eligible for the TPT but later did not receive the TPT, regardless of the reason, were defined as the non-TPT group. In this study, there were only a few people who did not fully comply with the treatment or complete TPT. They were excluded from the qualitative study to facilitate the representativeness of study results. The qualitative assessment was conducted only among the non-MBS and non-TPT groups.

Inclusion criteria for the qualitative part included being older than 18 years of age, and being able to communicate. We also attempted to ensure that participants were not under the influence of any narcotics, based on physical appearances and through informal questions. Nevertheless, no one was excluded due to that reason. Balance in gender, age distribution, occupation and proportionate samples from each village were also considered. A total of 30 individuals were included in the qualitative component of this research.

The data collection team was comprised of five members with university degrees who were trained by the researchers on data collection as well as ethical precautions for both quantitative and qualitative data collection. The team visited each household and collected census data from the household head or a representative. During in-depth interviews, one data collector would be the interviewee and the other would act as a note taker. The transcripts were written in the Burmese language. Each in-depth interview session lasts around 20 min.

### Data entry and analysis

The baseline population census was entered into a Microsoft Excel (Excel for Mac, Version 16.62) spreadsheet. Information regarding participation status, blood results and reasons for not being involved in TPT activity were recorded for each person. The charts were produced directly from the excel sheet. The data were then analyzed using the Statistical Package for the Social Sciences (IBM SPSS Statistics for Macintosh, version 23, IBM Corp., Armonk, NY, USA).

The quantitative component of this work began with a cross sectional survey that included a questionnaire and basic clinical assessments. The survey included data on age, gender, ethnicity, pregnancy and/or breastfeeding status, G6PD status, and mean hemoglobin levels. Exploratory statistical analyses were done to assess the characteristics of individuals who had G6PD deficiency and of those who did not participate in the study. Finally, a statistical analysis was done among all the eligible samples (n = 1315) to be involved in the TPT to explore sociodemographic factors relating to non-participation in the TPT. In this study, non-participation includes those who did not participate at all, or who only participated partially in the TPT, starting from initial blood screening to the completion of 14 days of PQ. Crude and adjusted odds ratios were reported together with 95% confidence intervals and p-values. All collected variables were entered into simple and multiple logistic regression models regardless of their statistical significance.

For the qualitative data, Burmese dialogs were entered in a Microsoft Excel file representing each respondent with an identity code and some general characteristics such as age, gender, and occupation. Each quote was then translated into English independently by PLA and MTS. KMW and MPK have ensured the accuracy and correctness of the translation. KO and DMP improved the English language and grammar. Finalized quotations were grouped under two situations: either non-participation in MBS or non-participation in TPT. The lead authors chose the most appropriate quotes, and other authors re-checked and agreed with the final ones.

### Ethics approval and consent to participate

The Institutional Review Boards of the University of Public Health, Yangon, Myanmar (UPH-IRB: 2020/ IR Research/ 2) and the University of South Florida, USA, reviewed and approved the study protocol. All methods were performed in accordance with the relevant guidelines and regulations. The respondents signed the informed consent forms before commencing the interviews and informed consent forms were obtained from all participants.

## Results

**Figure 2:**
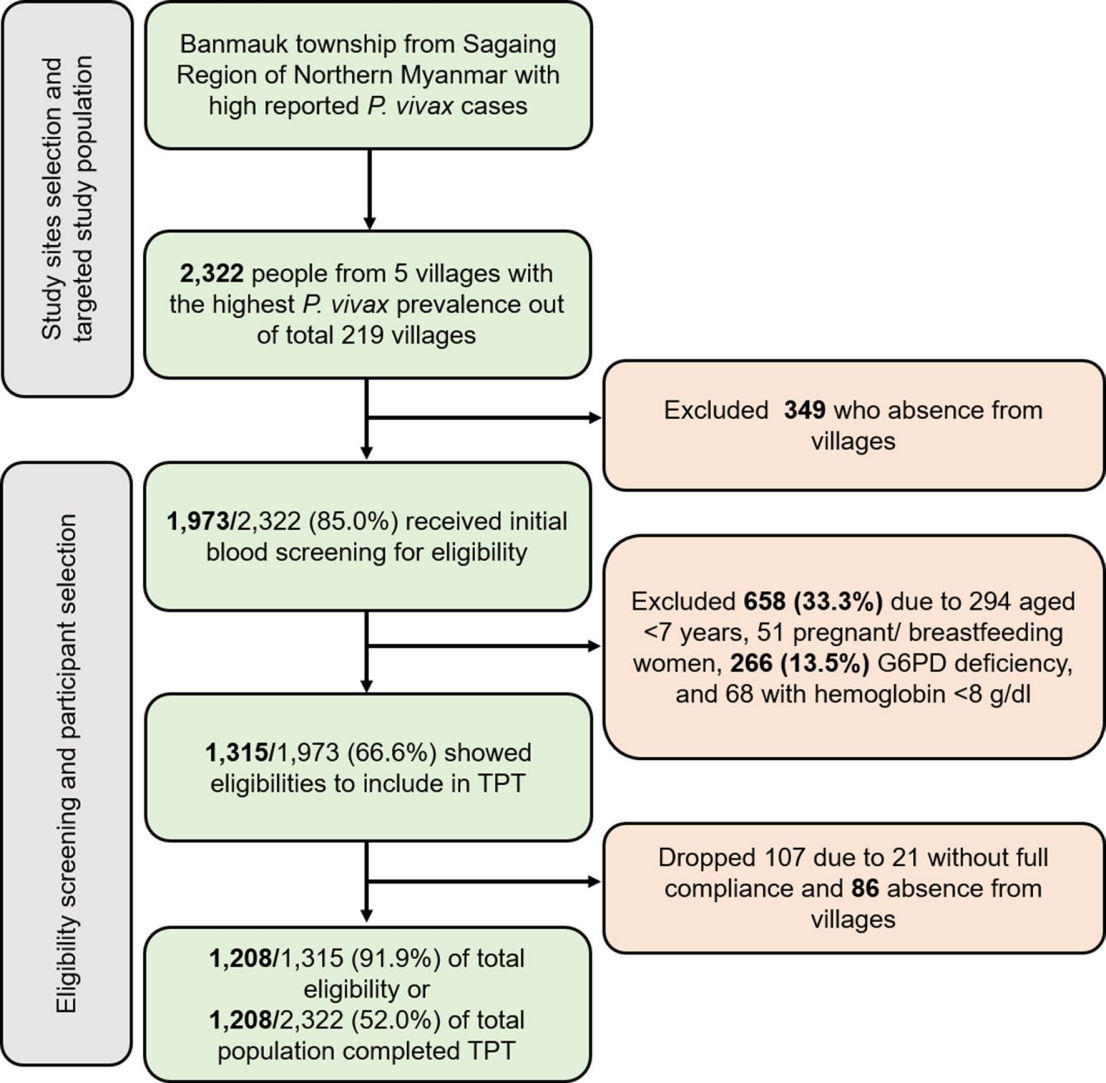
Schematic diagram for population screening for targeted primaquine treatment.

When implementing an initial mass blood survey (MBS), 349 individuals were away from the villages and missed the survey. Another 658 villagers were excluded because of the exclusion criteria, including 294 children who were < 7 years old, 51 women who were either pregnant or breastfeeding, 266 people who were G6PD deficient, and another 68 with Hb levels lower than 8 g/dl. The cumulative number was 658 because some individuals possessed both criteria (for example, a < 7-year-old folk could have G6PD deficiency too). This left a total of 1315 villagers who were eligible to be included in TPT. At the actual time of administration, a further 86 villagers were absent from the village and 21 individuals began but did not complete the radical cure regimen, leaving 1208 individuals who completed TPT. Overall, these final samples represented 52.0% of a total of 2322 villagers in the study villages, which was lower than the target set of overall 80.0% coverage. Still, it reached 91.9% of a total of 1,315 who were eligible to participate (Fig. 2).

### General characteristics of the targeted study population residing in each study village

The study villages varied by age, gender, and ethnic composition; as well as the proportion of villagers with G6PD deficiency (Table 1). One village (PintSinTe) had a younger population in comparison to the others. Three of the five villages had sex ratios that were skewed towards females (PayKone, ManKat, NantHnyin). Two villages were primarily Kadu ethnicity (PayKone and NaungKan), another two were primarily Shan ethnicity (ManKat and PintSinTe), and the last one was primarily Kanan ethnicity (NantHnyin). G6PD deficiency status differed by village as well, ranging from a low of 6.8% in PayKone to a high of 16.3% in ManKat.

**Table 1:** General characteristics of targeted study population residing in each study village (n = 1973).

General characteristics	PayKone (n = 207)	ManKat (n = 387)	PintSinTe (n = 393)	NantHnyin (n = 593)	NaungKan (n = 393)	Total
	n (%)	n (%)	n (%)	n (%)	n (%)	
Age (years)						
< 7	36 (17.4)	64 (16.5)	61 (15.5)	86 (14.5)	47 (12.0)	294 (14.9)
7–14	49 (23.6)	75 (19.4)	107 (27.3)	111 (18.7)	81 (20.6)	423 (21.4)
15–29	60 (29.0)	87 (22.5)	77 (19.6)	134 (22.7)	105 (26.7)	463 (23.5)
30–44	34 (16.4)	71 (18.3)	72 (18.3)	131 (22.1)	81 (20.6)	389 (19.7)
45–59	14 (6.8)	66(17.1)	56 (14.2)	75 (12.6)	49 (12.5)	260 (13.2)
≥ 60	14 (6.8)	24 (6.2)	20 (5.1)	56 (9.4)	30 (7.6)	144 (7.3)
Gender						
Male	74 (35.7)	146 (37.7)	181 (46.1)	232 (39.1)	209 (53.2)	842 (42.7)
Female	133 (64.3)	241 (62.3)	212 (53.9)	361 (60.9)	184 (46.8)	1131 (57.3)
Ethnicity						
Burmese	5 (2.4)	38 (9.8)	45 (11.5)	10 (1.7)	20 (5.1)	118 (6.0)
Kadu	197 (95.2)	–	–	–	373 (94.9)	570 (28.9)
Kanan	–	–	–	561 (94.6)	–	561 (28.4)
Shan	5 (2.4)	349 (90.2)	348 (88.5)	22 (3.7)	–	724 (36.7)
G6PD status						
Normal	193 (93.2)	324 (83.7)	344 (87.5)	499 (84.1)	347 (88.3)	1707 (86.5)
Deficient	14 (6.8)	63 (16.3)	49 (12.5)	94 (15.9)	46 (11.7)	266 (13.5)
Pregnancy/Breastfeeding						
Yes	3 (1.4)	10 (2.6)	16 (4.1)	18 (3.0)	4 (1.0)	51 (2.6)
No	204 (98.6)	377 (97.4)	377 (95.9)	575 (97.0)	389 (99.0)	1922 (97.4)
Hemoglobin value (g/dl)						
< 8	4 (1.9)	15 (3.9)	14 (3.6)	21 (3.5)	14 (3.6)	68 (3.4)
≥ 8	203 (98.1)	372 (96.1)	379 (96.4)	572 (96.5)	379 (96.4)	1905 (96.6)

### G6PD prevalence and its associated demographic factors among participants included in the initial mass blood survey

Among the total population (2322), 1973 (85.0%) were tested for G6PD status. Of 1973 samples, 266 (13.5%) were diagnosed as G6PD deficient. Their general characteristics were analyzed to find out possible associations with the presence of G6PD deficiency. Kadu, Kanan and Shan ethnic males living in ManKat, PintSinTe and NantHnyin villages were more likely to be G6PD deficient (Table 2).

**Table 2:** G6PD deficiency prevalence and its associated demographic factors among participants included in the mass blood survey (n = 1973).

Sociodemographic factors	G6PDd (n = 266)	G6PDn (n = 1707)	cOR (95% CI)	aOR (95% CI)	p
	n (%)	n (%)			
Gender					
Female	127 (11.2)	1,004 (88.8)	Ref	Ref	
Male	139 (16.5)	703 (83.5)	1.6 (1.21–2.03)	2.1 (1.63–4.09)	< 0.001*
Ethnicity					
Burmese	6 (5.1)	112 (94.9)	Ref	1	
Kadu	60 (10.5)	510 (89.5)	2.2 (0.93–5.21)	2.4 (1.50–7.11)	0.037*
Kanan	92 (16.4)	469 (83.6)	3.7 (1.56–8.58)	4.1 (2.23–10.2)	0.001*
Shan	108 (14.9)	616 (85.1)	3.3 (1.40–7.63)	3.6 (2.50–7.98)	0.003*
Living place (village)					
PayKone	14 (6.8)	193 (93.2)	Ref	Ref	
ManKat	63 (16.3)	324 (83.7)	2.7 (1.46–4.91)	2.8 (1.88–5.56)	< 0.001*
PintSinTe	49 (12.4)	345 (87.6)	2.0 (1.05–3.64)	2.0 (1.55–4.41)	0.017*
NantHnyin	94 (15.9)	499 (84.1)	2.6 (1.45–4.66)	2.9 (1.65–5.35)	< 0.001*
NaungKan	46 (11.7)	346 (88.3)	1.8 (0.98–3.42)	1.7 (0.99–3.31)	0.028*

*G6PDd* G6PD deficient, *G6PDn* G6PD normal, *cOR* crude odds ratio, *aOR* adjusted odds ratio, *CI* confidence interval, *Ref.* reference.

*Significance at p < 0.05.

Multivariable logistic regression showed that males (aOR: 2.1, 95% CI 1.63–4.09) were more prone to test positive for G6PD deficiency. Individuals from Kadu (aOR: 2.4, 95% CI 1.50–7.11), Kanan (aOR: 4.1, 95% CI 2.23–10.2) and Shan (aOR: 3.6, 95% CI 2.50–7.98) ethnic groups showed higher odds of being diagnosed with G6PD deficiency as well. There were also differences in G6PD deficiency prevalence among study villages. People residing in ManKat (aOR: 2.8, 95% CI 1.88–5.56), PintSinTe (aOR: 2.0, 95% CI 1.55–4.41), and NantHnyin (aOR: 2.9, 95% CI 1.65–5.35) villages had high prevalence of G6PD deficiency (Table 2).

### General characteristics of people who are absent from the villages during the mass blood survey and targeted primaquine treatment

A total of 435 (349 for the initial blood screening and 86 afterward) people were away from the villages during the TPT campaign. Most of them were male (64.4%), aged 15 to 29 years (47.4%), farmers (46.9%), annual family income was < 1,000,000MMK (61.6%), and those who had only completed primary (35.2%) or middle school (42.3%) levels. When they were asked for reasons for not being included in the TPT, more than one-third (43.0%) said they had stayed in farms, and a few (20.9%) mentioned that they lived in gold mine areas (Table 3**)**.

**Table 3:** General characteristics of people who were absent from the villages during mass blood survey and targeted primaquine treatment activity (n = 435).

Sociodemographic factors	n	%
Age (years)		
< 7	48	11.0
7–14	48	11.0
15–29	206	47.4
30–44	82	18.9
45–59	33	7.6
≥ 60	18	4.1
Gender		
Male	280	64.4
Female	155	35.6
Occupation		
Unemployed	31	7.1
Student	92	21.2
Farmers	204	46.9
Gold miners	101	23.2
Others (Teachers, Monks, Merchants)	7	1.6
Annual family income (MMK)		
< 1,000,000	268	61.6
1,000,000–1,999,999	147	33.8
2,000,000–2,999,999	14	3.2
≥ 3,000,000	6	1.4
Education		
No education/Small children	52	12.0
Primary school (Grade 1–5)	153	35.2
Middle school (Grade 6–9)	184	42.3
High school (Grade 10–11)	32	7.4
Above high school	14	3.2
Reason for not being in the villages		
Job-related or personal traveling	62	14.3
Attending school at other places	48	11.0
Staying in farms	187	43.0
Living in goldmine areas	91	20.9
No specific reason	47	10.8

1USD˜2100MMK.

### Reported side effects of 14-day primaquine treatment during targeted primaquine treatment

There was a total of 1208 participants who completed 14-day primaquine treatment during TPT. Among them, 94.0% reported no side effects while 6.0% presented minor symptoms. Side effects included dizziness (32 cases, 2.6%), headache (18 cases, 1.5%), epigastric pain symptoms (11 cases, 0.9%), palpitation (6 cases, 0.5%) and the last six cases (0.5%) revealing nausea and vomiting. To monitor for hemolysis, in addition, all participants were strictly followed up for dark colored urination. However, no one revealed presence of dark colored urine during the 14-day PQ treatment (Fig. S1; Table S1).

### Sociodemographic factors for non-participation among eligible samples in targeted primaquine treatment

The logistic regression indicated that three variables were significantly associated with non-participation in the TPT. First, participants in the 15 to 44 year old age range showed higher odds of not participating in the TPT when compared to the younger age group (aOR: 4.6, 95% CI 2.79–9.15) and (aOR: 2.4, 95% CI 1.19–4.66) for age groups (15–29 years) and (30–44 years), respectively. Second, males (aOR: 2.4, 95% CI 1.67–3.58) were more likely to not participate in the TPT. Third, farmers (aOR: 1.9, 95% CI 1.23–4.12) and gold miners (aOR: 3.5, 95% CI 2.21–6.11) possessed the highest odds of non-participation when compared to students. Annual family incomes and level of education were not associated with participation in the TPT in our analyses (Table 4).

**Table 4:** Underlying sociodemographic factors for non-participation of eligible samples in targeted primaquine treatment (n = 1315).

Sociodemographic factors	Non-TPT (n = 107)	TPT (n = 1208)	cOR (95% CI)	aOR (95% CI)	**p**
	n (%)	n (%)			
Age (years)					
7–14	15 (4.0)	357 (96.0)	Ref	Ref	
15–29	47 (15.7)	252 (84.3)	4.4 (2.43–8.11)	4.6 (2.79–9.15)	< 0.001*
30–44	26 (8.7)	274 (91.3)	2.3 (1.17–4.35)	2.4 (1.19–4.66)	0.007*
45–59	9 (4.2)	205 (95.8)	1.0 (0.45–2.43)	0.8 (0.23–2.55)	0.459
≥ 60	10 (7.7)	120 (92.3)	2.0 (0.87–4.53)	1.8 (0.67–3.90)	0.052
Gender					
Female	48 (6.0)	749 (94.0)	Ref	Ref	
Male	59 (11.4)	459 (88.6)	2.0 (1.35–2.99)	2.4 (1.67–3.58)	< 0.001*
Occupation					
Student	20 (5.1)	370 (94.9)	Ref	Ref	
Unemployed	3 (5.9)	48 (94.1)	1.2 (0.33–4.04)	1.1 (0.21–4.03)	0.410
Farmers	68 (9.2)	672 (90.8)	1.9 (1.12–3.13)	1.9 (1.23–4.12)	0.008*
Gold miners	16 (12.7)	110 (87.3)	2.7 (1.35–5.37)	3.5 (2.21–6.11)	0.002*
Annual family income (MMK)					
< 1,000,000	54 (7.4)	677 (92.6)	0.9 (0.45–1.74)	0.7 (0.35–1.45)	0.361
1,000,000–1,999,999	31 (10.1)	277 (89.9)	1.2 (0.60–2.55)	1.1 (0.55–2.18)	0.278
2,000,000–2,999,999	11 (7.7)	132 (92.3)	0.9 (0.39–2.21)	0.9 (0.33–2.20)	0.430
≥ 3,000,000	11 (8.3)	122 (91.7)	Ref	Ref	
Education					
High school (Grade 10–11)	5 (9.3)	49 (90.7)	Ref	Ref	
No education	7 (14.6)	41 (85.4)	1.7 (0.49–5.67)	1.5 (0.38–5.67)	0.204
Primary school (Grade 1–5)	58 (7.0)	773 (93.0)	0.7 (0.28–1.92)	0.7 (0.23–1.91)	0.265
Middle school (Grade 6–9)	36 (10.0)	325 (90.0)	1.1 (0.41–2.90)	1.1 (0.42–3.11)	0.435
Above high school	1 (4.8)	20 (95.2)	0.5 (0.05–4.46)	0.4 (0.03–4.63)	0.263

1USD˜2100MMK.

Other occupation groups including 3 teachers, 2 monks and 3 merchants were involved entirely in TPT and excluded from logistic regression.

*TPT* targeted primaquine treatment, *cOR* crude odds ratio, *aOR* adjusted odds ratio, *CI* confidence interval, *Ref.* reference.

*Significance at p < 0.05.

## Qualitative findings

### Among the non-MBS group

Although many villagers were aware that the TPT campaigns had health benefits at both individual and community-levels, they did not participate in the campaign. Most people who missed the initial blood screening reported being absent because they traveled to worksites such as farms and goldmines to find a better income for their families (many were heads of their respective households). In addition, a few high school students could not participate in the TPT because they had to live and study at their high schools away from their villages.

“I realized we should adhere to the healthcare intervention which is very important to control malaria. However, in my family, I am the only one who earns a sufficient income by being employed in a gold mine. Thus, I had to work and stay in the workplace. Therefore, I could not participate in the initial mass blood screening.” (38-year-old man, non-MBS).

“My family completed all treatment except two of my children who missed receiving the blood test and taking the drug because they lived in Banmauk township to attend grade-9 and -10 classes. I informed them about the TPT campaign during a monthly visit to their schools, but their return to the village was impossible.” (46-year-old man, non-MBS).

“We have heard about giving anti-malarial treatment to all people in our villages. However, most of our family could not return to the village as the time coincided with our cultivation period and when the roads were terrible.” (28-year-old woman, non-MBS).

“In my family, 4 out of 6 members joined this mass treatment campaign. The other two (my husband and elder son) went to another township to seek new job opportunities to solve our family’s current financial difficulties.” (39-year-old woman, non-MBS).

“I just came back from the goldmine 2 days ago. I have been notified that there was a mass treatment activity in this village. However, I could not return to the village to participate in the activity because of my responsibility for ongoing mining activities.” (31-year-old man, non-MBS).

### Among the non-TPT group

Some people were eligible to receive the treatment, but they had to work in forested areas far from the village. Some expressed that they will indeed participate once there is another round of mass treatment. Many people know the potential advantages of TPT, but they were afraid of the consequences if they could not adhere to the full treatment course. Most people also know the TPT program is free of charge.

“I already had my blood tested (for G6PD deficiency) and was told I am eligible to receive antimalarial treatment. However, I had to visit my native town during the proposed days when the campaign was occurring. Hence, I did not get any treatment. If there is another chance, I am sure I will participate.” (28-year-old woman, non-TPT).

“I had a chance to participate in the first mass blood survey organized by the research team. I was also informed of the drug treatment schedule. But the proposed time frame coincides with our cultivation time, and I have missed a chance to get the treatment, unfortunately.” (42-year-old woman, non-TPT).

“I was informed that the malaria team will provide me 14-days of primaquine treatment under their observation as I am eligible to participate; however, while waiting, the manager called me to come into a gold mine. Therefore, I did not receive the treatment as I am afraid of adverse consequences of incomplete treatment.” (48-year-old man, non-TPT).

“To be honest, I am interested in this mass treatment activity as this is the first activity ever to reduce the malaria burden in our village without any charges. However, I have to work in a gold mine for my family's income, and I missed the opportunity to participate in it.” (35-year-old woman, non-TPT).

## Discussion

Interventions such as mass administration of antimalarials may provide both individual and population-level benefits^19,25,28^, but require high levels of community participation in order to achieve the population level benefits (though most research on the needed coverage levels has focused on falciparum malaria). Achieving high levels of community participation can be challenging for many reasons. In this analysis, we identify innate un-modifiable community characteristics that lead to non-participation, as well as other characteristics that may be addressed either through community engagement or through adaptations by the TPT implementing teams.

Individuals with G6PD deficiency may experience severe side effects if they take PQ^6,19,29^, and were therefore excluded from this study. The evidence base with regard to use of PQ in pregnancy and while breastfeeding is scant. Testing for G6PD deficiency status for in utero fetuses is difficult, invasive, and rarely done and PQ administration for pregnant women (regardless of their G6PD status) is often contraindicated^6,30^. Some recent evidence suggests that PQ levels in breastmilk are quite low, but PQ administration in lactating women is still rare^31^. Finally, in this study children < 7 years were excluded from the targeted treatment with PQ based on recommendations from collaborators at Mahidol University and following Thai National guidelines for trials. Thus, almost one-third of all people who participated in the blood screening in these study villages were excluded due to having one or more of these exclusion criteria. Mitigation of some inclusion and exclusion criteria (e.g. expanding the age range to less than 1 year to be included in the TPT) might lead to increased coverage of TPT.

G6PD deficiency is not a modifiable attribute, and in this study we found that the proportions of G6PD deficient individuals varied by study village, by ethnicity, and by gender. This is an important non-modifiable attribute that should be considered when planning mass administration of PQ, as a very high proportion of G6PD deficiency could make it impossible to achieve targeted coverage of PQ in a given community. The reported G6PD deficiency in Myanmar ranges between 10.0% to 20.0%^16,32,33^. However, currently, 14-day PQ has been prescribed by healthcare providers including basic health staff and volunteers without G6PD testing^6^. Patients with *P. vivax* are instructed to regularly check their urine color during the treatment and if there is dark-colored urine or any unusual anemic symptoms, they should come back to the health facility^6^. In Myanmar, people living in malaria-endemic areas with a history of taking PQ have rarely reported any hemolysis features.

The G6PD-normal TPT participants in this study also reported no severe side effects of PQ during the treatment. G6PD screening by qualitative or quantitative methods is recommended in future trials. Based on the quantitative results, including non-severe and severe forms of G6PD deficiency in the TPT could be considered with different treatment regimens, which is also in line with the current NMTG^6^. Mass treatment with a shorter regimen (e.g. 7 days of treatment) or the 8-week PQ regimen (in case of mild or moderate G6PD deficiency) may also avoid loss of compliance or increase coverage^16,34,35^. Given that Shan, Kadu, and Kanan ethnic males had a higher risk of G6PD deficiency, they should be closely monitored with access to regular hemoglobin checks and emergency medical care in upcoming TPT operations.

There were several other factors that were associated with non-adherence in this study, many of which could plausibly be addressed through community engagement or through modifications among the TPT teams. For example, working-age people showed a higher chance of non-participation in TPT. In the study villages, family incomes have mainly relied on agricultural-related or gold mining activities usually employed by working age groups. This group of people is responsible for their respective family's economic conditions and several reported needing to work rather than participate in healthcare activities including TPT. This finding is in line with a previous study^24^ done in this setting. Therefore, healthcare support and social development programs should come together in later trials to facilitate the involvement of working-age individuals. For example, mobile healthcare workers might be able to administer the treatment at farms or other work sites. Delivering DOT by family members rather than healthcare providers might be an alternative for villagers who usually work in the daytime and come back home at nighttime^36^.

In Myanmar culture, males are often accountable for family matters especially with regard to income. Work needs often conflict with health awareness-raising activities. Some studies have documented poor levels of malaria knowledge and practices among males when compared to females^37,38^. Therefore, targeted health education should be delivered to the male group to persuade their interest to participate in future TPT campaigns, along with potential modifications to the actual administration of PQ.

Likewise, Myanmar is an agricultural country and many people living in the nation earn their living from farm-related activities^39^. Typically, they cultivate their nearby farms around the villages once the rains begin, which often corresponds with the malaria season. Many of them work in rural and remote areas, and roads and other infrastructure need to be developed across the country. While there have been improvements over the last decade, the country is in the developing stage amid an economic crisis impacted by COVID-19 pandemic and political affairs. Therefore, during the rainy season many people have to stay in farming areas as road conditions have deteriorated and travel is difficult. Consequently, farmers are often outside the purview of healthcare coverage, which normally focuses on villages and communities, and they experience a high risk of malaria infection especially during the rainy season when malaria transmission is high^40^.

Similarly, gold miners often must stay near mines (often in forested settings) day and night. Therefore, they could not come back to their households. Findings from the qualitative component also corroborated this. Thus, in the next TPT campaigns, implementing mass treatment activities in the dry season or setting up malaria volunteers at the worksite might solve this challenge^41^. Implementing TPT activities inside or nearby goldmines through malaria volunteers might lead to good TPT coverage. Otherwise, these groups of missing people might serve as an ongoing reservoir and source of malaria transmission. A study^22^ concluded that one of the reasons hampering the attainment of full coverage for mass treatment was migration of the targeted participants. There are a few published articles on TPT activities solely targeting forest-related workers and this area of work should be further pursued. Antimalarial chemoprophylaxis with ACT^42^ or PQ^43^ also has a potent prophylactic effect to prevent malaria transmission. Targeting high-risk groups such as gold miners in the TPT campaign might help increase overall population coverage. Moreover, malaria preventive practices and treatment-seeking behaviors among forest-related workers have historically been considerably low^44,45^. Therefore, malaria education should be carried out as well as surveillance systems to detect newly emerged cases.

There are both strengths and limitations to this study. To our knowledge, this study was the first research exploring the overall feasibility of primaquine mass treatment in Myanmar. As part of the study, the prevalence of G6PD deficiency was estimated for this township and it can also be applicable for considering routine G6PD testing in the national malaria surveillance system in the future. In addition to quantitative data, the inclusion of qualitative data added to our understanding of the processes under study, especially with regard to the lived challenges and barriers to participating in TPT. There are also a few limitations to this study. The study villages were selected because of the burden of vivax malaria. Our analysis is representative of these study villages, but may not be representative of others. Likewise, these data were collected in late 2019, prior to the onset of the COVID-19 pandemic and the recent political troubles in Myanmar. The healthcare and healthcare administration context has drastically changed. While there are still practical and useful lessons that have emerged from this study, there are likely new challenges that will need to be overcome if targeted mass treatment with PQ is to be implemented in Myanmar.

## Conclusions

There were several potentially useful lessons-learned from this study, perhaps especially with regard to future PQ trials in Myanmar or other similar regions. Only half of the targeted population completed the TPT after excluding ineligible participants and community members who were absent for part or all of the study (e.g. those who were away from the villages for work). An alternative approach like giving a shorter PQ regime during the dry season may increase adherence among some participants. In addition, implementing targeted treatment activity around the field sites or inside the workplaces by DOT may be a good option. Mitigating some inclusion criteria (e.g. including the 1- to 7-year-old children into the TPT) may be considered in upcoming trials to achieve higher overall coverage. Strengthening community awareness of targeted treatment activities especially among working-aged male farmers and gold miners might increase participation.

## Supplementary Information

Supplementary Table S1.

Supplementary Figure S1.

## Data Availability

All data generated or analyzed for this study are available from the corresponding authors upon reasonable request.

## Abbreviations

aOR: Adjusted odds ratio
CI: Confidence interval
cOR: Crude odds ratio
DOT: Directly observed treatment
GMS: Greater Mekong Subregion
G6PD: Glucose 6 phosphate dehydrogenase
MBS: Mass blood survey
NMTG: National malaria treatment guidelines
TPT: Targeted primaquine treatment
VBDC: Vector borne disease control
WHO: World health organization
