# Supplementary material for: Factors hindering coverage of targeted mass treatment with primaquine in a malarious township of northern Myanmar in 2019–2020
Source: Sci Rep. 2023 Apr 12;13:5963. doi: 10.1038/s41598-023-32371-4 (PMC10091336; doi:10.1038/s41598-023-32371-4)
Supplement: Supplementary file 1 — Supplementary Table S1. [file 41598_2023_32371_MOESM1_ESM.docx]

**Table S1**. Reported side effects of 14-day primaquine during targeted primaquine treatment (n=1,208).

| **Symptom** | **n** | **%** |
| --- | --- | --- |
| **Side effects** | | |
| No reported side effects | 1135 | 94.0 |
| Dizziness | 32 | 2.6 |
| Headache | 18 | 1.5 |
| Epigastric pain | 11 | 0.9 |
| Palpitation | 6 | 0.5 |
| Nausea and vomiting | 6 | 0.5 |
