# Supplementary material for: Factors hindering coverage of targeted mass treatment with primaquine in a malarious township of northern Myanmar in 2019–2020
Source: Sci Rep. 2023 Apr 12;13:5963. doi: 10.1038/s41598-023-32371-4 (PMC10091336; doi:10.1038/s41598-023-32371-4)

**Supplementary Figure 1**: Reported side effects of 14-day primaquine during targeted primaquine. treatment (TPT). A) Side effects of 14-day primaquine among all TPT participants. B) Breakdown of participants who experienced side effects from primaquine treatment.


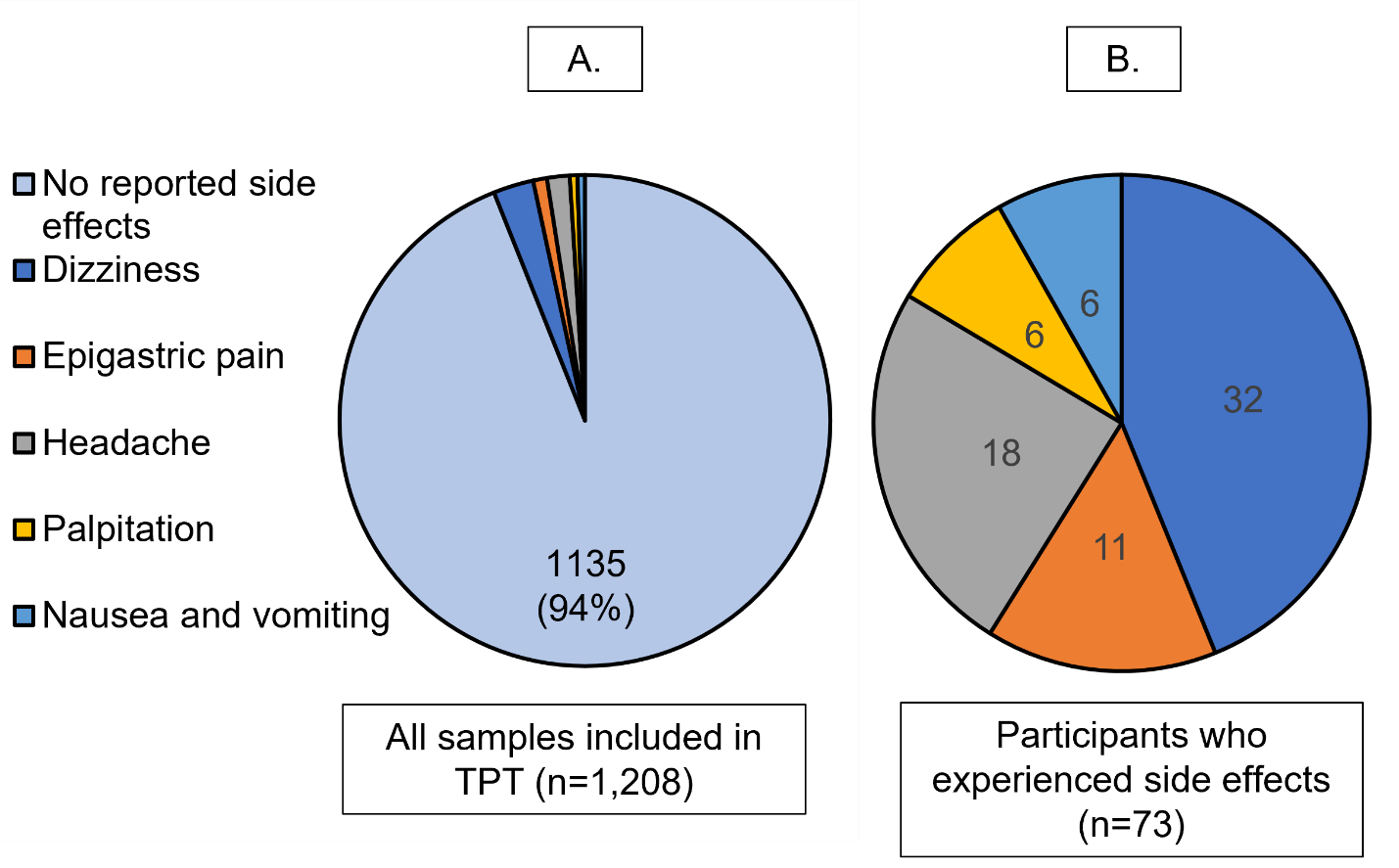

Supplement: Supplementary file 2 — Supplementary Figure S1. [file 41598_2023_32371_MOESM2_ESM.docx]
